# Solazyme: "unlocking the power of microalgae: a new source of sustainable and renewable oils"

**DOI:** 10.1186/1753-6561-8-S4-O38

**Published:** 2014-10-01

**Authors:** Alda Lerayer

**Affiliations:** 1 SOLAZYME, Campinas, São Paulo, Brazil

## 

• **The technology platform of Solazyme:** Solazyme´s biotechnology platform transforms a wide variety of sugars into renewable oils with high added value, using microalgae, microorganisms that are naturally capable of storing energy in the form of oil. Solazyme's technology enables the conversion of this feedstock into oils for a variety of applications including chemical, cosmetic, food and fuel. Unique to Solazyme's process is their proprietary ability to tailor oils, meaning that for the first time in the history, Solazyme has the ability to design oil profiles rather than simply use what's available in nature today. Through its world class tailoring capabilities, Solazyme is coupling proprietary strains of algae with standard industrial biotechnology; converting what the earth produces naturally- carbohydrate sugars- into what society needs most - oil.

• **The existing source of oils × new source of oil**: While the physical and chemical characteristics of conventional oils have traditionally been dictated by oils found in nature, or blends derived from them, this is no longer the case. Solazyme has created a new paradigm that enables the company to design and produce novel tailored oils that cannot be achieved through blending of existing oils alone. This core competency has created an incredible market opportunity. Solazyme´s platform enables the production of renewable oils with different carbon chain lengths, saturation levels and profiles simply by changing the microalgae strain used in the fermenter.

• **Solazyme renewable oils - the microalgae**: Through Solazyme's industrial biotechnology platform, the company is able to harness the oil-producing capability of microalgae, an organism that has evolved naturally to produce oil prolifically and efficiently, making it an ideal organism for industrial fermentation. The company's technology allows for the optimization of oil profiles with different carbon lengths, saturation levels and functional groups to modify important oil characteristics, such as flash point, pour point, cloud point, oxidative stability, smoke point, and viscosity, thus enabling much more stable and sustainable end products. Solazyme's platform is also feedstock flexible and can utilize a wide variety of renewable plant-based sugars, such as sucrose, dextrose, and sugar from other sustainable biomass sources including cellulosics. Utilizing standard industrial fermentation equipment to efficiently scale and accelerate microalgae's natural oil production time to a few days, Solazyme feeds their proprietary, oil-producing microalgae plant-based sugars in enclosed fermentation tanks, where they are in effect utilizing "indirect photosynthesis," in contrast to the traditional open-pond approach.

• **Markets:** Solazyme leverages their proprietary biotechnology platform to tailor oils that address major markets served by conventional oils: transportation fuels, chemicals, skin and personal care products, nutritionals, among many others.

• **Sustainability and safety - breaking paradigms:** Solazyme oils address many of the challenges associated with traditional oils, such as supply constraints, volatile pricing, and potentially negative and irreversible environmental effects. The "drop-in" nature of Solazyme's tailored oils enables compatibility with existing production, refining, and distribution infrastructures in each of its target markets. Solazyme´s algal oil are fully traceable back to their source of origin and are more sustainable than any other plant-based or petroleum-based oil. They are produced locally and therefore environmental and logistic costs are lower; feedstock comes from the sugar/alcohol industry, a closed carbon system; Solazyme's oil has the lowest GHG profile as compared to any other plant-based and petroleum-based oils.

